# Quasi-Simultaneous Sensitive Detection of Two Gas Species by Cavity-Ringdown Spectroscopy with Two Lasers

**DOI:** 10.3390/s21227622

**Published:** 2021-11-17

**Authors:** Guosheng Ma, Yabai He, Bing Chen, Hao Deng, Ying Liu, Xingping Wang, Zhihao Zhao, Ruifeng Kan

**Affiliations:** 1 Anhui Institute of Optics and Fine Mechanics, Hefei Institutes of Physical Sciences, Chinese Academy of Sciences, Hefei 230031, China; gsma@aiofm.ac.cn (G.M.); yabaihe@hotmail.com (Y.H.); bchen@aiofm.ac.cn (B.C.); hdeng@aiofm.ac.cn (H.D.); yliu@aiofm.ac.cn (Y.L.); 2 Graduate School of Science Island Branch, University of Science and Technology of China, Hefei 230026, China; 3 School of Engineering Science, University of Science and Technology of China, Hefei 230026, China; wxp048@mail.ustc.edu.cn; 4 College of Information Science and Engineering, Northeastern University, Shenyang 110819, China; 1970813@stu.neu.edu.cn

**Keywords:** cavity ringdown spectroscopy, optical sensing, simultaneous detection of multi components, methane isotope ratio

## Abstract

We developed a cavity ringdown spectrometer by utilizing a step-scanning and dithering method for matching laser wavelengths to optical resonances of an optical cavity. Our approach is capable of working with two and more lasers for quasi-simultaneous measurements of multiple gas species. The developed system was tested with two lasers operating around 1654 nm and 1658 nm for spectral detections of ^12^CH_4_ and its isotope ^13^CH_4_ in air, respectively. The ringdown time of the empty cavity was about 340 µs. The achieved high detection sensitivity of a noise-equivalent absorption coefficient was 2.8 × 10^−11^ cm^−1^ Hz^−1/2^ or 1 × 10^−11^ cm^−1^ by averaging for 30 s. The uncertainty of the high precision determination of $\delta^{13}{\mathrm{CH}}_{4}$ in air is about 1.3‰. Such a system will be useful for future applications such as environmental monitoring.

## 1. Introduction

Methane (CH_4_) is an important biosignature gas that provides key clues for the existence of life on extraterrestrial planets, such as Mars [1]. Methane is also an important greenhouse gas [2], energy resource [3], and microbial metabolites product [4] in ambient air. The processes of CH_4_ production and consumption can be understood through the analysis of its isotopes, so as to obtain CH_4_ source information. For example, more negative $\delta^{13}C$ and δD values indicate biological sources, while more positive $\delta^{13}C$ and δD values indicate heat sources [5].

One type of widely used instrument for measuring trace gases and isotopes is isotope ratio mass spectrometry (IRMS) with an accuracy of ±0.2‰ for δCH413 [6]. However, its in-situ application is limited due to its large size, high cost and complicated operation process [7]. In recent years, with the development of diode lasers, such as those used in fiber-optical tele-communication and longer wavelength interband and quantum cascade lasers, laser-spectroscopy-based sensors have become a more attractive tool for trace gas detection and its isotopes analysis. It offers high sensitivity, small size, fast response, and high selectivity [8,9,10,11,12,13,14,15] in spectroscopic gas sensing. In particular, cavity ringdown spectroscopy (CRDS) can achieve an effective absorption path length of tens of kilometers by using a high-finesse optical cavity, which greatly improves the detection capability of trace gas sensing [16]. CRDS has been extensively applied in atmospheric greenhouse gas detections [17,18,19], aerosol particle extinction measurements [20,21], spectral line parameters measurements [22,23,24], and human breath diagnosis [25,26,27]. Because of the high sensitivity of CRDS, it is very suitable for high-precision stable isotope analysis. One early CRDS methane isotope measurement was in the mid-infrared wavelength region, due to the stronger absorption line intensity in the mid-infrared region. Dahnke et al. measured the absorption of ^12^CH_4_ and ^13^CH_4_ near 3 μm, and its measurement precision for $\delta^{13}C$ in ambient air was ±11‰ [28]. With the development of longer wavelength quantum cascade lasers, it became possible to apply CRDS on the strong fundamental vibrational transitions of ^12^CH_4_ and ^13^CH_4_ at 7.5μm, as reported by Abhijit Maity et al. for δ13CH4 measurements in the atmosphere and human breathing [29]. In the near-infrared region, the line intensity of methane is about two orders of magnitude weaker than that in the mid-infrared region. However, much higher reflectivity mirrors available for the near-infrared region increase the effective optical path length of absorption, which can largely compensate for weaker line intensity. In addition, near-infrared lasers and optical components are cheaper and more robust; hence, trace gas and isotope measurements based on near-infrared (NIR) continuous-wave cavity ringdown (CW-CRDS) have attracted attention. Chen et al. used a pair of high-reflection mirrors exceeding 99.9993% to build a CRDS system with an equivalent absorption optical length of 93.3 km and achieved high-precision measurements of δ13CH4 in the air [30]. A commercial instrument made by Picarro is also based on NIR CW-CRDS. In addition to being used in ambient air [31], it also provides insights into the aquatic carbon cycle process [32].

This paper reports the development of a CRDS instrument which is capable of high-precision measurements of two species quasi simultaneously. The present instrument operates with dual lasers at wavelengths of ~1654 nm for ^12^CH_4_ and ~1658 nm for isotope ^13^CH_4_ measurements in this work. In the following sections, we will provide a detailed description of the system and an application demonstration of the system for isotope ratio measurements of CH_4_ in air.

## 2. Experimental System

**Figure 1:**
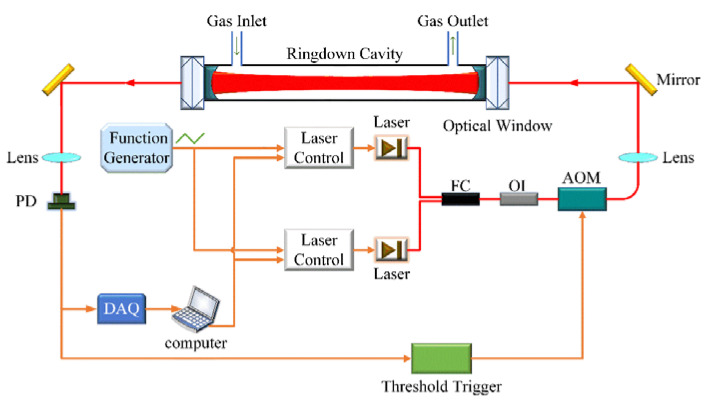
Schematic of the experimental setup with two lasers. FC, fiber-optic combiner; OI, optical isolator; AOM, acousto-optic modulator as an optical switch; PD, photodiode detector; DAQ, data acquisition.

The schematic of our developed system is presented in Figure 1. Two near-infrared distributed feedback (DFB) diode lasers (from NEL) were utilized for spectral detections of ^12^CH_4_ and its isotope ^13^CH_4_ in wavelength regions of 1654 nm and 1658 nm, respectively. Two laser drivers (SRS, model LDC501) were used to control the operation temperatures and currents of the two diode lasers. These operation parameters were adjustable by a computer via a GPIB interface. The laser wavelength can be quickly modulated via its operation current by a triangle-waveform signal from a function generator (Siglent, model SDG1032X). Both laser outputs were combined by a fiber combiner and then passed through an optical isolator (FOPTO, model PIISO-1654-D-L-05-FA), which is used to block the light back-reflected by the ringdown cavity from entering the laser and to minimize any influence of the optical feedback effect on the laser stability. An acousto-optic modulator (Brimrose, model AMM-55-8-70-1630-2FP) is used to switch off the 1st-order deflected laser beam into the cavity during the measurements of ringdown signals. The input laser beam geometry was mode-matched to the longitudinal modes of the optical cavity by using a lens.

The optical ringdown cavity consisted of a pair of highly reflective plano-concave mirrors (Layertec, reflectivity > 99.995%, radius of curvature 1000 mm). The cavity was sealed by separate window quartz substrates at both ends, so that the cavity mirrors will not experience any differential pressure stress for better stability. The cavity was vacuumed and heated to around 200 °C for 48 h to desorb the gas adsorbed on the inner wall of the cavity before setting up the system. A high-vacuum turbo pump (Pfeiffer, model HiCube 80 Eco) was used to extract the gas in the cavity to a pressure as low as 10^−3^ Pa.

The transmitted optical signal from the ringdown cavity was collected into a single- mode optical fiber by a lens and received by a photodetector (Femto, model LCA-S-400K-IN-FS). Based on a preset threshold level for the transmitted signal, a trigger signal was generated to drive the AOM to turn off the 1st-order deflected laser beam and obtain a ringdown process of the signals. No noticeable impact on the ringdown decay time was observed when the threshold was set at different levels. A 16-bit data acquisition device (NI, model USB-6356, 1.25 MS/s data sampling rate) was used for acquiring cavity ringdown signals.

## 3. Methods

### 3.1. Step-Scanning Laser Frequency for Achieving Cavity Optical Resonances

Optical resonance between input laser radiation and an optical cavity occurs when the round-trip optical path length of the cavity is approximately an integer multiple of the laser wavelength. Therefore, we could tune and dither the laser wavelength for achieving an optical resonance [33,34] instead of an active stabilization of the laser wavelength. During an optical resonance process, the low-power laser radiation will be effectively coupled through the highly-reflective mirror into the cavity so that subsequent cavity ringdown decay could be facilitated by switching off the input laser beam [35,36,37] or moving the laser wavelength off resonance with the cavity [11]. In this study, we use a fixed-length cavity and step-scan the laser wavelength in combination with a small wavelength dithering. The periodic resonance frequencies of a fixed-length cavity serve as an accurate frequency scale for the measurement spectra. The longitudinal mode frequencies of the ringdown cavity are:

(1) $$\nu_{N}=\nu_{0}+N\times FSR,$$

where $\nu_{0}$ is the offset starting frequency of the cavity mode, N is an integer number for the cavity mode, and *FSR* is the free spectral range (i.e., periodicity of the resonance frequencies) of the cavity.

As the CRDS cavity uses highly-reflective mirrors, the bandwidth of an optical resonance is very narrow (~kHz). The exact resonance frequency of the cavity is susceptible to mechanical vibrations and temperature fluctuations from the external environment. Furthermore, the stability of DFB-type diode lasers (as used in this work) is typically in the order of ~MHz. Therefore, we modulated the laser wavelength slightly to achieve the optical resonance. The magnitude of the modulation is about FSR/4. This will ensure that the laser radiation interacts with a unique cavity mode and does not interfere with an adjacent cavity mode during the spectral scanning process [38].

**Figure 2:**
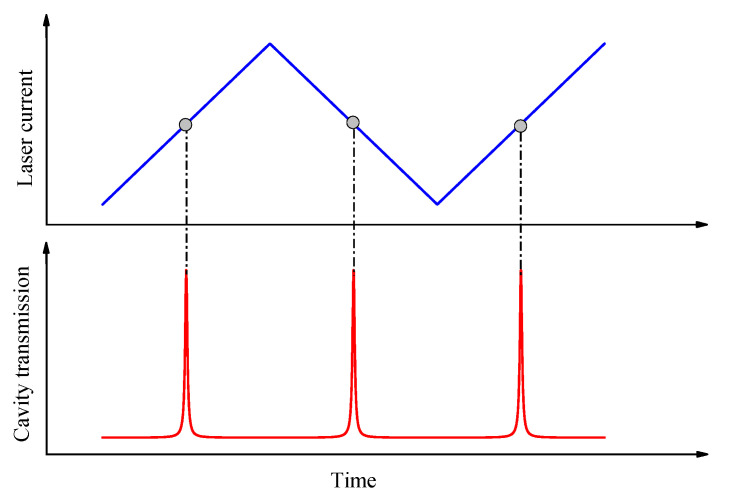
Mode matching the laser wavelength to the resonance of an optical cavity by feedback controlling the laser operation current so that the resonance occurs in the middle region of a small dithering of laser current.

**Figure 3:**
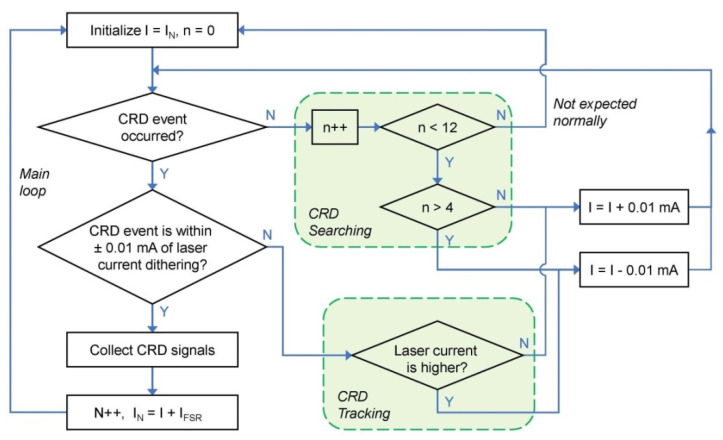
Flowchart of the control scheme for searching, tracking, and step-scanning the laser wavelength to match optical cavity resonances.

We can feedback control the laser wavelength by probing the position of a mode matching during the triangle dithering and by adjusting the laser operation current to create resonance around the middle region of dithering, as shown in Figure 2. After multiple ringdown events have been measured, the laser current is step advanced by an amount corresponding to a laser frequency change in the vicinity of the consecutive cavity resonance, and the cycle is repeated until a preset range of spectral points has been acquired. The flow chart of this laser wavelength control scheme is shown in Figure 3. The steps on the left-hand side in Figure 3 form the main loop. The tracking loop is for centering the laser wavelength onto cavity resonances, whereas the searching loop is for moving the laser current until a CRD event happens within the triangle dithering. The temperature of the laser is maintained constant to an accuracy of ~1 mK. The triangular signal for current dithering was set at ~100 Hz, with an amplitude for making a laser frequency change of about 1/4 of the FSR. This small modulation amplitude ensures that the laser interacts with only one longitudinal cavity mode at a time and ringdown events can occur efficiently.

**Figure 4:**
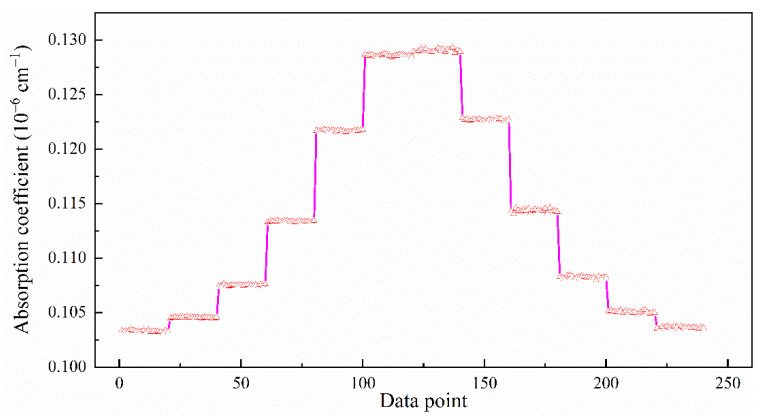
Recording of a step-scanned spectrum across an absorption line of ^13^CH_4_ at 1658.689 nm. At each laser frequency set to a consecutive cavity resonance frequency, 20 ringdown events were measured.

In order to verify and demonstrate the feasibility of the scheme, a spectrum was measured that continuously recorded 20 ringdown events at each scan step of the laser wavelength for consecutive cavity resonance frequencies. Figure 4 shows a step-scanned absorption feature of ^13^CH_4_ at 1658.689 nm, demonstrating that the laser frequency scan and CRDS measurements over an absorption feature were reliable.

### 3.2. Simultaneous Multi-Wavelength Operation with Two or More Lasers

A single DFB diode laser has a limited wavelength coverage range. Therefore, multiple lasers might be required for spectral measurements of different absorption features or different gas species [39]. One approach is to use an optical switch for the selecting light output from one of the multiple laser sources. In this paper, we report a new method. We track the wavelengths of all the lasers relative to the cavity resonance frequencies of a fixed-length cavity and bring only one laser at a time to resonance with the cavity while all the other lasers are kept off-resonance with the cavity. The highly-reflective cavity mirrors block all the light of the non-resonant lasers. Therefore, the detectable cavity transmission is from a single laser only.

**Figure 5:**
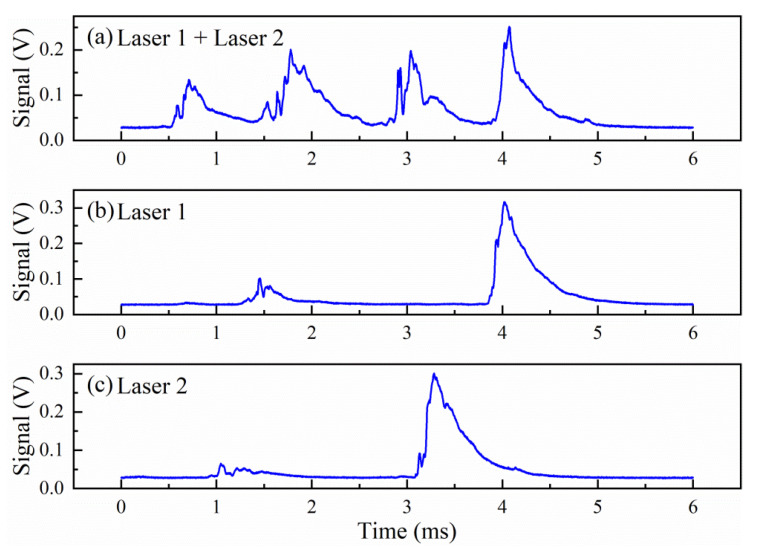
(**a**) Ringdown signals when both lasers are in resonance with the cavity within a short period of time, causing interference. (**b**) and (**c**) Ringdown signal when only one laser is dithered around cavity resonance while the other laser is set off resonance with the cavity. The signal fluctuation is caused by frequency fluctuation of the DFB diode lasers.

Figure 5 shows cavity transmission with two lasers. When both laser frequencies are modulated simultaneously around cavity resonance, cavity transmission signals interfere with each other, as shown in Figure 5a, and the ringdown signals can overlap. By keeping one laser off resonance and modulate only one of the lasers across the cavity resonance, a clear single-wavelength ringdown event can be created and measured, as shown in Figure 5b,c. We used the wavelength tracking scheme explained before and shown in Figure 3 to identify the operation conditions for each of the lasers at resonance with the cavity. For setting lasers off resonance with the cavity, we offset its operation current corresponding to a frequency shift of FSR/2. Quasi simultaneous detection at two or more different wavelengths is achieved by alternating one laser on resonance and all the other lasers off resonance with the cavity.

## 4. Results and Discussion

### 4.1. Measurements of the Cavity’s FSR Serve as a Frequency Scale of Spectral Scans

**Figure 6:**
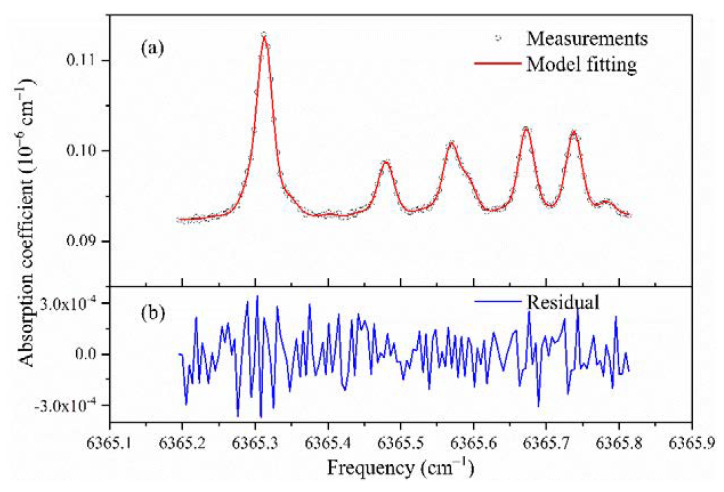
(**a**) A CH4 absorption spectrum measured at discrete frequency points of cavity resonances, together with spectral modelling. (**b**) The established CH4 wavelengths help to determine the cavity mode spacing (i.e., its free spectral range—FSR).

The FSR value of an optical cavity is related to its optical path length. By directly measuring the cavity length, one can estimate its FSR. For example, a two-mirror cavity of 0.5 m length has an FSR of ~300 MHz. In practice, the error of such estimation is relatively large. A better way of determining the FSR is to simultaneously record a reference spectrum of well-known transition frequencies and the cavity transmission. The frequency interval and the corresponding number of cavity resonances enable the determination of the FSR via a straight-line fitting of Equation (1). By using widely separated spectral features, the accuracy of FSR can be improved further as needed. This process is illustrated in Figure 6. The absorption spectral frequencies of CH_4_ in the range of 6365.19–6365.81 cm^−1^ are from the HITRAN2016 database. The choice of this wavelength range is based on the fact that there are multiple absorption peaks in a smaller wavelength range, and it is within the operation range of the diode laser used.

**Figure 7:**
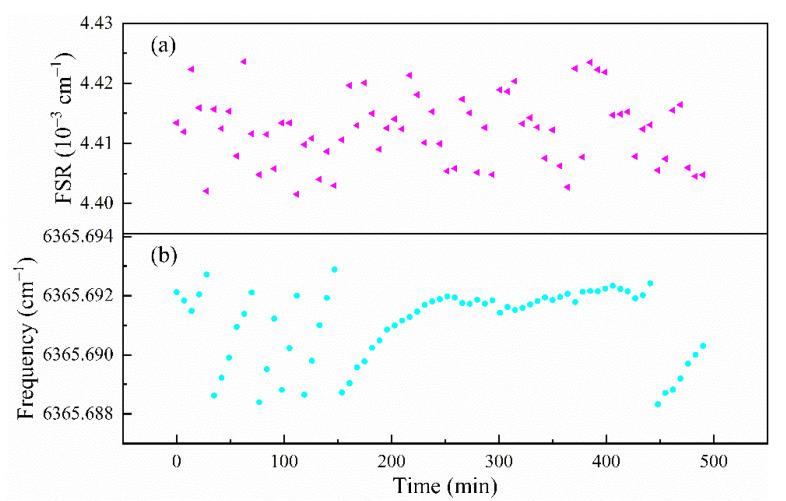
(**a**) Repeated measurements of the FSR value of the ringdown cavity and (**b**) the slow drift of one of the cavity resonance frequencies as a result of optical cavity length drift. The large step change of about 0.0044 cm^−1^ is due to the FSR periodicity in resonance frequency assignment.

We repeated such measurements many times for 500 min. The FSR values and the slow drift of one cavity resonance frequency (i.e., cavity length) are displayed in Figure 7. The estimated average FSR value was (4.412 ± 0.006) × 10^−3^ cm^−1^, and the corresponding cavity length was 113.33 ± 0.15 cm. The good matching between the measurement spectrum and its modeling (see Figure 6) confirms that there are no missing spectral data points in the step-scanned spectrum over the cavity resonances. Figure 7b shows a drift of the cavity length and the associated exact resonance frequencies which varied during the 500 min (~8 h), as they were affected by the variation of room and cavity temperature. Please note that the large step change of about 0.0044 cm^−1^ (one FSR) is due to a shift in mode assignment to an adjacent longitudinal mode. As each spectrum is recorded within a short time, and only the spacing (FSR) between the spectral data points is relevant, the slow drift of the cavity has no impact on spectral measurements of gas concentrations.

### 4.2. Instrument Sensitivity

**Figure 8:**
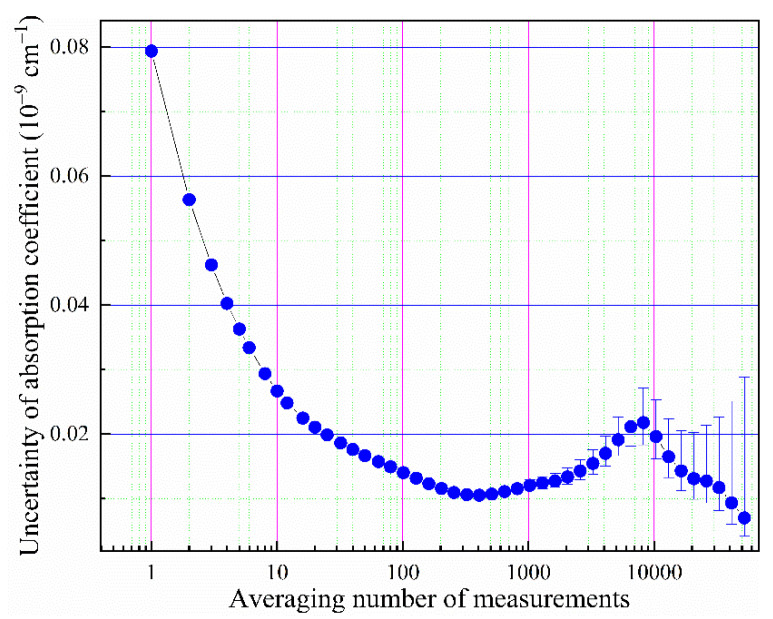
Allan variance analysis of the noise-equivalent absorption coefficient of the empty cavity as a function of the number of ringdown measurements, together with indications of 95% confidence intervals. The data rate is 9 measurements per second.

The detection sensitivity, or the minimum detectable absorption coefficient ($\alpha_{\min}$), of the system is usually characterized by the noise-equivalent absorption coefficient (NEA), which is the smallest change in the absorption coefficient that can be detected in a unit time [40]. Averaging over time could reduce the detection noise, until the drift in detection becomes dominant [41]. Allan variance analysis is a useful tool for evaluating the long-term stability of the system, its detection limit, and its optimal integration time [42]. We evaluated the system performance by continuous measurements at one resonance frequency of the empty ringdown cavity evacuated by a turbopump. The ringdown time of the empty cavity was about 340 µs. As shown in Figure 8, the Allan variance plot shows that the optimal average number CRDS measurements is 270 times, which corresponds to the integration time of 30 s. The data rate was 9 measurements per second. The NEA of the system is estimated to be 2.8 × 10^−11^ cm^−1^ Hz^−1/2^ (at the position of 9 measurements in Figure 8) and $\alpha_{\min}$ is 1×10^−11^ cm^−1^ after reaching the optimal integration time of 30 s (at the position of ~270 measurements in Figure 8).

### 4.3. Large Range of Absorption Measurements

**Figure 9:**
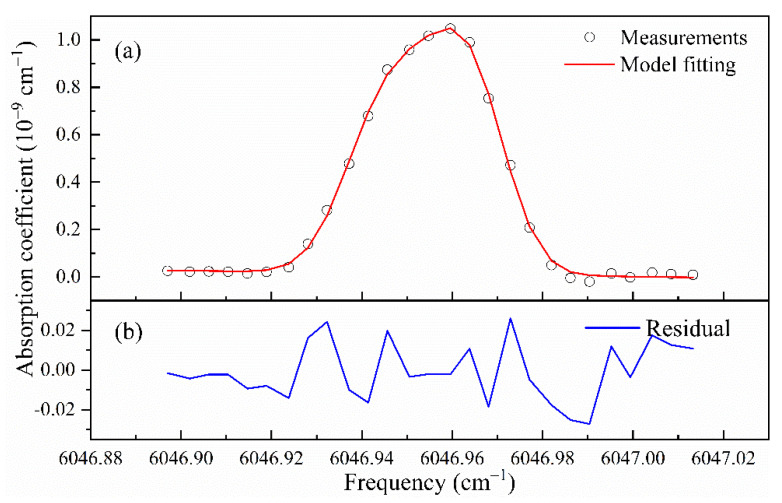
(**a**) Trace CH_4_ measurements near 6046.95 cm^−1^ and (**b**) fitting residuals.

**Figure 10:**
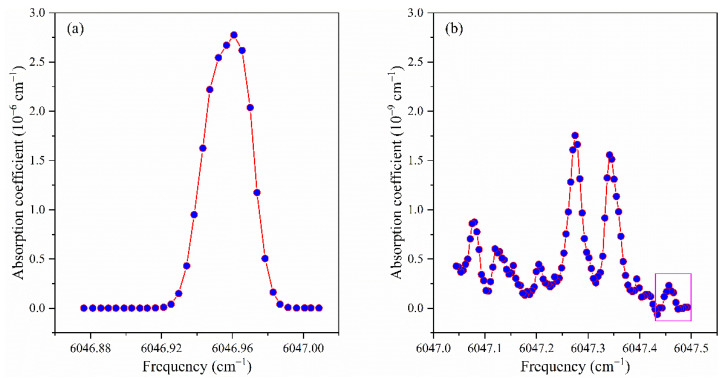
Spectral measurements around (**a**) 6046.95 cm^−1^ and (**b**) 6047.30 cm^−1^, with an estimated CH_4_ partial pressure of 0.1 pa. The vertical scale of (**b**) is 1000 times smaller than that of (**a**).

The trace CH_4_ detection capability of the system is proved by measuring the residual CH_4_ in the cavity when the cavity is flowed with CH_4_ gas sample and continuously evacuated to maintain the pressure in the cavity at 0.07 pa. Such a measurement result is shown in Figure 9. The partial pressure of methane in the cavity is estimated to be 4.55 × 10^−5^ pa by fitting the measured absorption spectrum of three unresolved CH_4_ absorption lines. The residual noise level indicates that the detection limit of methane partial pressure is less than 10^−6^ pa. Figure 10 shows the measured spectrum of methane with an estimated partial pressure of 0.1 pa when the total pressure of CH_4_ and N_2_ in the cavity is 1 pa. The small rectangular box in Figure 10b shows that the absorption coefficient corresponding to this absorption line is in the order of 10^−10^ cm^−1^. This demonstrates that the instrument is capable of measuring a large range of absorption coefficients from ~10^−6^ cm^−1^ to ~10^−10^ cm^−1^. By selecting absorption features of different line strengths, the instrument could cover a large concentration range of CH_4_ measurements.

### 4.4. CH_4_ Isotope Analysis

The abundance of stable isotopes is usually expressed by the value of the parameter δ, which is defined as the deviation from the value of the international reference standard Vienna Pee Dee Belemnite (*VPDB*) for carbon isotopes:

(2) δ13C=((CH413/CH412)sample(CH413/CH412)VPDB−1)×1000 ‰.

**Figure 11:**
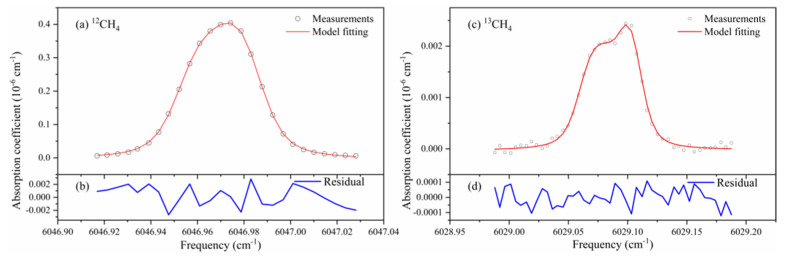
Spectral measurements of (**a**) ^12^CH_4_ and (**c**) ^13^CH_4_ isotope in ambient air, together with model fittings. (**b**) and (**d**) are fitting residuals.

**Figure 12:**
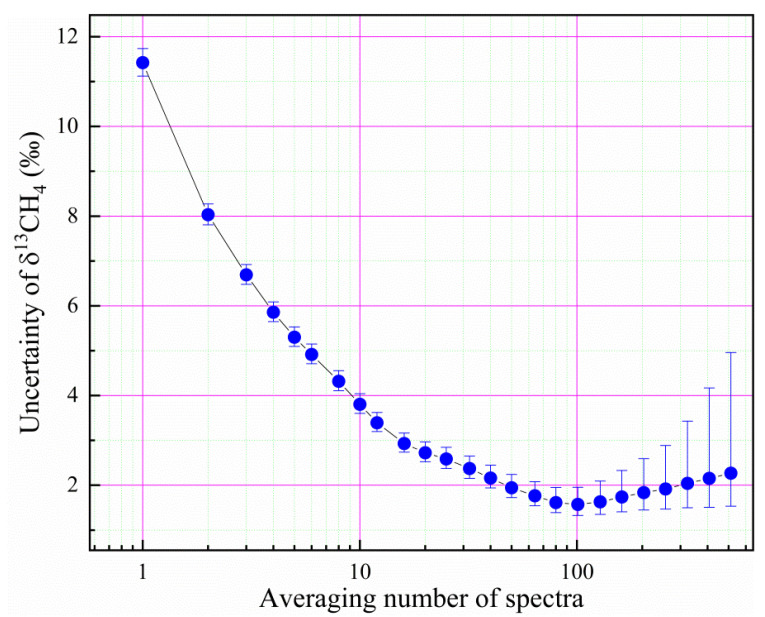
Allan variance analysis for the precision of CH_4_ isotope measurements, together with indications of 95% confidence intervals.

The ^12^CH_4_ has an absorption feature in the near infrared region around wavelength 1.6537 µm (or frequency 6046.98 cm^−1^), whereas the isotope ^13^CH_4_ has its absorption feature around 1.6586 µm (or frequency 6029.10 cm^−1^). We have employed two DFB-type diode lasers for their spectral detections, respectively. Figure 11 shows measurement spectra of ^12^CH_4_ and ^13^CH_4_ in ambient air using the dual-wavelength detection method presented in the previous sections. The pressure in the cavity was controlled at 10,000 pa and the temperature was stabilized at 299 ± 0.01 K. Each data point of the ^12^CH_4_ spectrum in Figure 11a was the result of a single ringdown event, and the much weaker ^13^CH_4_ spectrum averages 20 ringdown events into one data point in Figure 11b. We repeated such spectral measurements 2000 times and performed an analysis of Allan deviation on δCH413 values, as shown in Figure 12. The results indicate that the instrument is capable of a precision of 1.3‰ on δCH413 by averaging the measurement results of 100 spectra. One factor that affects the precision of isotope measurement was a large temperature gradient (0.2 K) in the cavity. This was based on the temperature difference measured between the two ends of the cavity. It is expected to achieve higher accuracy if the temperature uniformity was better.

## 5. Conclusions

This paper presents a CRDS system that combines a scheme for matching laser wavelengths to optical cavity resonances and dual-laser spectral measurements. A scanning algorithm is applied to step-scan and track the laser wavelength to the optical cavity resonance. In order to avoid the mutual interference of ringdown signals between the two lasers, we operated the laser coupling to the cavity in a time-multiplex fashion. The highly reflective cavity mirrors helped to block off non-resonant lasers. We determined the free spectra range (FSR) of the fixed-length cavity by counting the steps of a step-scanned CH_4_ spectrum with known spectral line positions in the region of 6365.19–6365.81 cm^−1^. The FSR interval serves as a frequency scale of measurement spectra. The $\alpha_{\min}$ and NEA of the system are 1 × 10^−11^ cm^−1^ and 2.8 × 10^−11^ cm^−1^ Hz^−1/2^, respectively. By selecting absorption features of different strengths, measurements can detect a wide range of methane concentrations. The dual-laser measurements (1654 nm and 1658 nm) achieved a high-precision determination of atmospheric δCH413 (1.3‰). This optical instrument will be useful for future environmental monitoring applications and can be extended for other gas species by using additional lasers operating at different wavelengths. A recent innovative development exploited quartz tuning forks (QTFs) as a photodetector based on a light-induced thermo-elastic effect [43]. It offers advantages of high sensitivity and no wavelength limitation for future optical measuring systems, as reported for an ultra-high sensitive detection of carbon monoxide using a mid-infrared quantum cascade laser [44].
